# Effects of particulate matter (PM) on childhood asthma exacerbation and control in Xiamen, China

**DOI:** 10.1186/s12887-019-1530-7

**Published:** 2019-06-13

**Authors:** Jinzhun Wu, Taoling Zhong, Yu Zhu, Dandan Ge, Xiaoliang Lin, Qiyuan Li

**Affiliations:** 1 Department of Pediatrics, the First Affiliated Hospital of Xiamen University, No.55 Zhenhai Road, Xiamen, 361003 China; 2 National Institute for Data Science in Health and Medicine, School of Medicine, Xiamen University, South Xiang’an Road, Xiamen, 361102 China

**Keywords:** Childhood asthma, Particulate matter, Exacerbation, Asthma control, Electronic health record;Xiamen

## Abstract

**Background:**

The short-term effects of particulate matter (PM) exposure on childhood asthma exacerbation and disease control rate is not thoroughly assessed in Chinese population yet. The previous toxic effects of PM exposure are either based on long-term survey or experimental data from cell lines or mouse models, which also needs to be validated by real-world evidences.

**Methods:**

We evaluated the short-term effects of PM exposure on asthma exacerbation in a Chinese population of 3106 pediatric outpatientsand disease control rate (DCR) in a population of 3344 children using case-crossover design. All the subjects enrolled are non-hospitalized outpatients. All data for this study were collected from the electronic health record (EHR) in the period between January 1, 2016 and June 30, 2018 in Xiamen, China.

**Results:**

We found that exposure to PM_2.5_ and PM_10_ within the past two weeks was significantly associated with elevated risk of exacerbation (OR = 1.049, *p* < 0.001 for PM_2.5_and OR = 1.027, *p* < 0.001 for PM_10_). In addition, exposure to PM_10_ was associated with decreased DCR (OR = 0.976 for PM_10_, *p* < 0.001).

**Conclusions:**

Our results suggest that exposure to both PM_10_ and PM_2.5_ has significant short-term effects on childhood asthma exacerbation and DCR, which serves as useful epidemiological parameters for clinical management of asthma risk in the sensitive population.

**Electronic supplementary material:**

The online version of this article (10.1186/s12887-019-1530-7) contains supplementary material, which is available to authorized users.

## Background

Asthma is a chronic allergic respiratory disease with a heterogeneous background involving both genetic and environmental factors. In 2016, 339.4 million people worldwide were affected by asthma [1]. In China, the prevalence of asthma was 3.02% in children under 14 years old (95%CI:2.97–3.06%) [2]. Corticosteroids therapy can relieve the symptoms of asthma, however, the prevalence of asthma still increased significantly over the past 20 years [3, 4]. Exposure to all ergens in the pollutants is one of the major risk factors of asthma in children [5]. Evidence currently available has shown that many environmental factors, including allergens, airborne irritants, unfavorable weather conditions and adverse indoor environment, are associated with asthma progression [6, 7]. Inhalable particulate matter (PM) including PM_2.5_ and PM_10_ (inhalable particles with an aerodynamic diameter less than or equal to 2.5 μm and 10 μm, respectively), is known as major environmental hazardous factors that impact human health [8–11]. Previous epidemiological studies have shown that high concentrations of PM_2.5_ and PM_10_ are associated with elevated mortality rate and increased incidence of many diseases, such as respiratory diseases, cardiovascular diseases, central nervous system diseases and inflammation [12–14]. In China, PM has become a major cause of air pollution due to rapid industrialization and urbanization in recent years [15, 16]. This fact leads to growing concerns on the part of hospitals, government and the public about the health risks associated with PM. In particular, the ability of stakeholders to predict the impact of PM on public health is essential for hospitals to take timely and efficient actions to handle overwhelming outpatient volume caused by hazardous environmental conditions.

Most of the studies conducted worldwide addressed the relationship between PM exposure and asthma in terms of long-term effects and few assessed the impact of PM exposure on asthma control rate [17].Many studies address the transient effect of indoor and ambient pollutants and allergens on asthma exacerbation but less is known for particulate matters [18, 19]. On the other hand, current empirical studies examining the toxic effects of PM exposure were mostly conducted in cell lines and mouse models based on case-control design. Real world evidence derived from electronic health record (EHR) is likely to provide more pragmatic and accurate estimate of the effects of PM exposure [20, 21].

It has been well documented that PM exposure causes specific immune responses in the airway [22–24]. PM induces inflammation, apoptosis, increased secretion of T-cell cytokines, and DNA damage [25, 26]. Asthmatic symptoms are documented in 14% of children worldwide [27]. Children are more susceptible to PM-related diseases because of higher breathing rates, narrower airways, immature lung tissue, and longer exposure time to outdoor ambient air [28, 29].

Xiamen is located on the southeast coast of China. The city is in typical subtropical climate zone. No study is available to address the short-term effects of PM on childhood asthma exacerbation and control rate in this area. Considering the increasing PM pollution in this area and growing public health concern over PM, there is a need to obtain further epidemiological evidences for public health service to take proper preventive measure to control the risk caused by PM exposure. Therefore,we designed this study to evaluate the effects of PM exposure on childhood asthma exacerbation and control rate.

## Methods

### Patient data

Childhood asthma data were collected from the electronic health record system of Pediatric Outpatient Department of the First Affiliated Hospital of Xiamen University (Joint Commission International accredited hospital). All subjects are outpatients between zero and 14-year-old, who were diagnosed with asthma exacerbation inthe period from January 1, 2016 to June 30, 2018.The diagnosis of childhood asthma is based on respiratory symptoms including wheezing, shortness of breath, chest tightness or cough (Additional file 1 Table 1). Patients with respiratory symptoms caused by other diseases were excluded. The classification of asthmafollowsthe International Classification of Disease 10 (ICD-10-CM) code of J45 [27]. The study was designed conforming to the ethical guidance (KY2015–027). For each case of acute exacerbation, the date of the latest asthma exacerbation was determined. Patients whose symptoms reappeared within 14 days were defined as the same one exacerbation, and the last exacerbation was selected as index exacerbation.

For asthma control, the outcome was determined upon return visit after four-week treatment since the initial visit based on Guidelines forthe Diagnosis and Treatment of Childhood Bronchial Asthma [30]. The outcome of the disease is defined for children aged below and above six separately (Additional file 1 Table 2). We further classify the cohort in to two subgroups, well-controlled asthma and uncontrolled or partly controlled asthma [31, 32]. Asthma was managed with budesonide aerosol inhalation, fluticasone MDI with spacerdevices, or budesonide or budesonide/formoterol powder in halation according to patients’ age. The patients were followed up every oneto three months. In case of acute exacerbation, salbutamol aerosol or budesonide and aerosolized terbutaline solution for inhalation were added. Appropriate treatment was added if there was comorbidity, such as allergic rhinitis or infection. The outcome of asthma was assessed according to “Guidelines for the Diagnosis and Prevention of Asthma in Children” [30]. Disease control was rated as well controlled, partly controlled, or uncontrolled according to the daytime and night symptoms inthe past 4 weeks.

### Air pollution data

Air pollution data were obtained from Xiamen Department of Environmental Protection. The concentration of pollutants was measured at different sites of the city. Daily average PM_10_ and PM_2.5_ concentrations were used to measure the exposure. Meteorological data including daily average ambient temperature,wind speed, cumulative precipitation, humidity and barometric pressure were obtained from Xiamen Meteorological Bureau.

### Statistical analysis

**Fig. 1:**
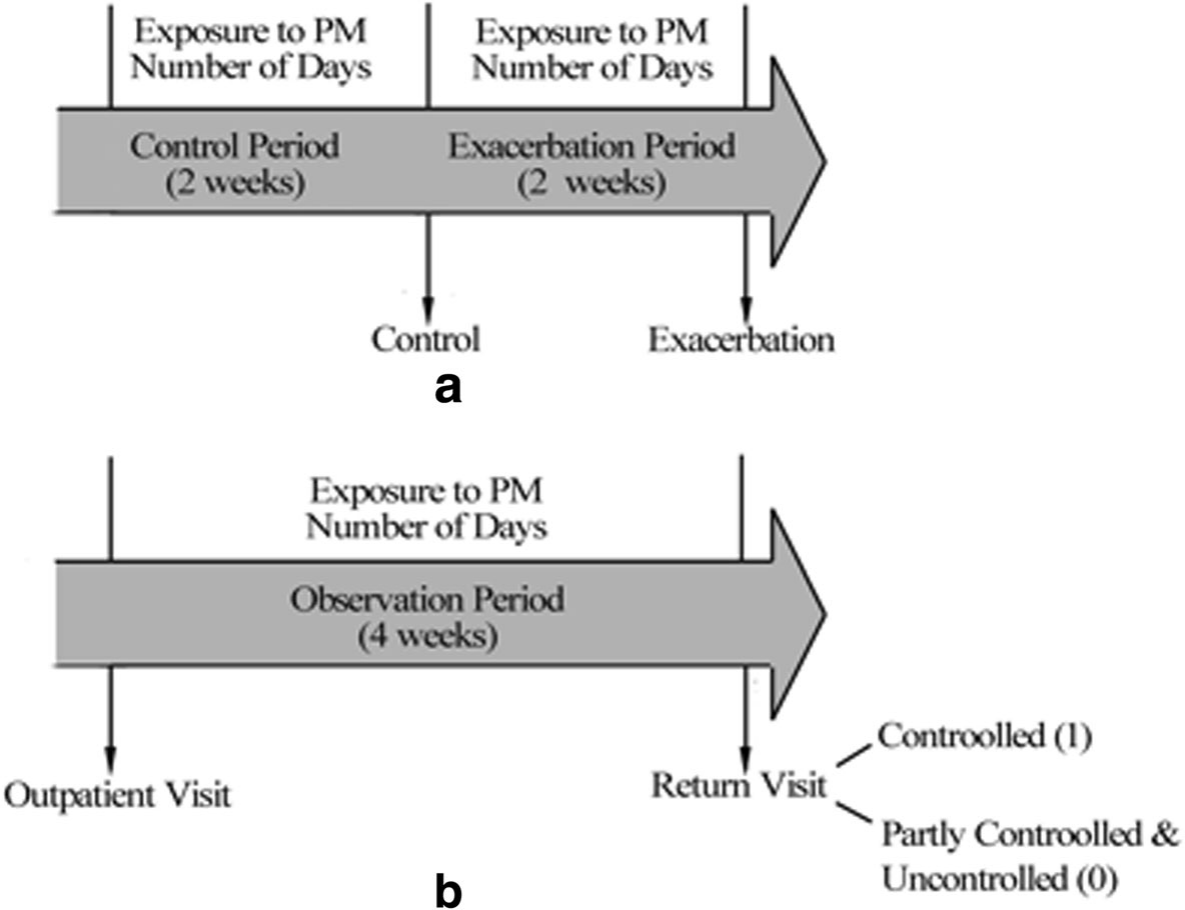
Schematic view of the study design. Panel (**a**):For patients of acute exacerbation, the day two weeks before the exacerbation was considered as control. The PM exposures within 2 weeks before the exacerbation day and control day were recorded,respectively. Panel (**b**):The PM exposure within 4 weeks before the return visitwas recorded. Patients were assessed at follow-up visit based on the symptoms in the past 4 weeks

Case-Crossover (CCO) designwas used to assess the effects of PM on asthma exacerbation. To measure the exposure to PM, we recorded the number of days of AQI (air quality index) level 2 or 3(24-h average of PM_2.5_ > 35 μg/m^3^ and PM_10_ > 50 μg/m^3^) [33] within two weeks preceding the onset of the index exacerbation(Fig. 1a). We also measured the four-week-exposure before the time point of control evaluation for rating disease control. The outcome of disease control was defined as 1 if asthma was controlled, or 0 if the disease was partly controlled or uncontrolled(Fig. 1b).

To evaluate the effects of PM exposure on asthma exacerbation and control rate, mixed effects logistic regression was performed,in which PM exposure was considered as a fixed effect and individual patient as random effects. Fever and weather conditions including average temperature, cumulative precipitation and average wind speed were covariates in the model. We standardized the estimated odds ratio (OR) for each fixed effect to compare the effects of different factors.

The model is described as:

$$ \mathrm{logit}\left(\mathrm{P}\right)=\log \left(\frac{\mathrm{P}}{1\hbox{-} \mathrm{P}}\right)=\upbeta \ast \mathrm{M}+\uptau \ast \mathrm{T}+\upgamma \ast \mathrm{R}+\upomega \ast \mathrm{W}+\upvarphi \ast \mathrm{F}+\upmu \ast \mathrm{s} $$

where, P is the probability of asthma exacerbation or control, M is the measure of exposure of PM_2.5_ or PM_10_; s is a random grouping variable corresponding to each individual; T is average temperature, R is cumulative precipitation, W is average wind speed and F is fever. β, τ, γ, ω, φ and μ are regression coefficients.

As there is high collinearity between PM_10_ and PM_2.5_, which is evidenced by pairwise Pearson correlation coefficient of 0.906, which would lead to instability in effect estimates in multivariate regression analysis, the regression models were built with the two air pollutants separately.

All statistical procedures were conducted using R-3.5.

## Results

### Summary of patient information

A total of 3106 patients with 4728 cases of acute asthma exacerbation were identified from 16,355 cases of childhood asthma (Table 1). The patients included 2110 (67.9%) males and 996 females (32.1%). The age of these patients ranged from zero to fourteen years old. Patients aged four to six accounted for the largest proportion (39.9%), showing that preschool children were affected by asthma mostly. In the control period, 53 patients (1.1%) in the study had fever and during the 2 weeks before exacerbation there were 832 patients (18.2%) who had fever. Among the 3443 returning-visit patients, 2292 (66.6%) were males and 1151 (33.4%) were females, and children aged four to six accounted for the largest proportion (44.8%). In the course of the 4 weeks in which we assessed the control level of the patients, 6.6% of the patients had fever. There are nine subtypes of J45 present in the cohort (Additional file 2 Figure 1a). Bronchial asthma (J45.903) makes the majority of the cohort (41.2%), followed by asthmatic bronchitis (J45.901, 26.2%) and cough variant asthma (J45.005, 19.7%). The other subtypes (J45.004, J45.900, J45.000, J45.904, J45.006 and J45.003) cover 12.9% of the cohort.

**Table 1:** Patients’ characteristics of the study cohorts

		Number	Percentage
Gender	Male	2110	67.90
	Female	996	32.10
Age(years)	0–3	1230	39.60
	4–6	1238	39.90
	7–14	638	20.50
	Total	3106	100
Return visit			
Gender	Male	2292	66.60
	Female	1151	33.40
Age(years)	0–3	1127	32.70
	4–6	1542	44.80
	7–14	774	22.50
	Total	3443	100

### Summary of the exposure measures and covariates

**Fig. 2:**
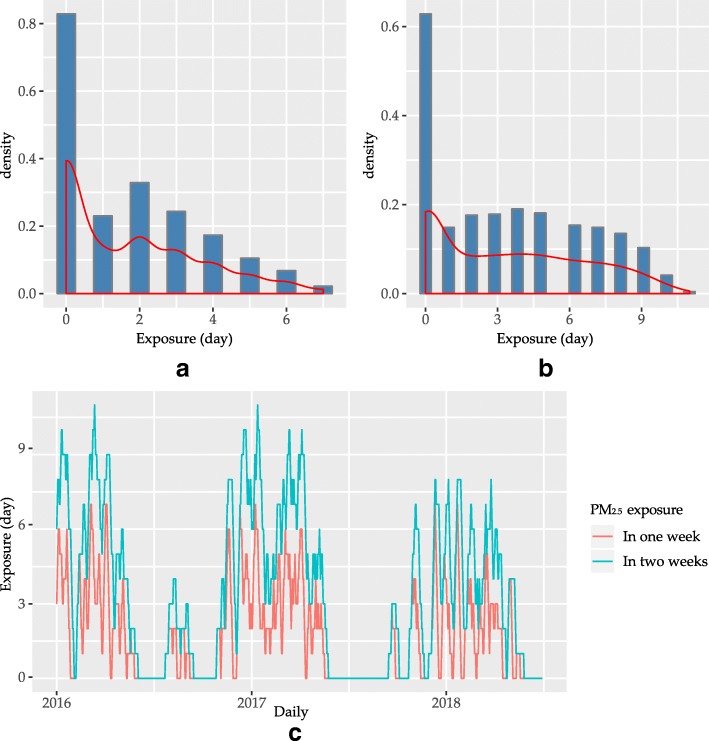
Distribution and exposure leveltoPM_2.5_. Panel(**a**): Distribution of exposure days of PM_2.5_ in one week before the exacerbation and the density curve;Panel(**b**): Distribution of exposure days of PM_2.5_ in two weeks before the exacerbation and the density curve; Panel(**c**): Exposure days of PM_2.5_ in one week (red line) and in two weeks (blue line) before the exacerbation

**Fig. 3:**
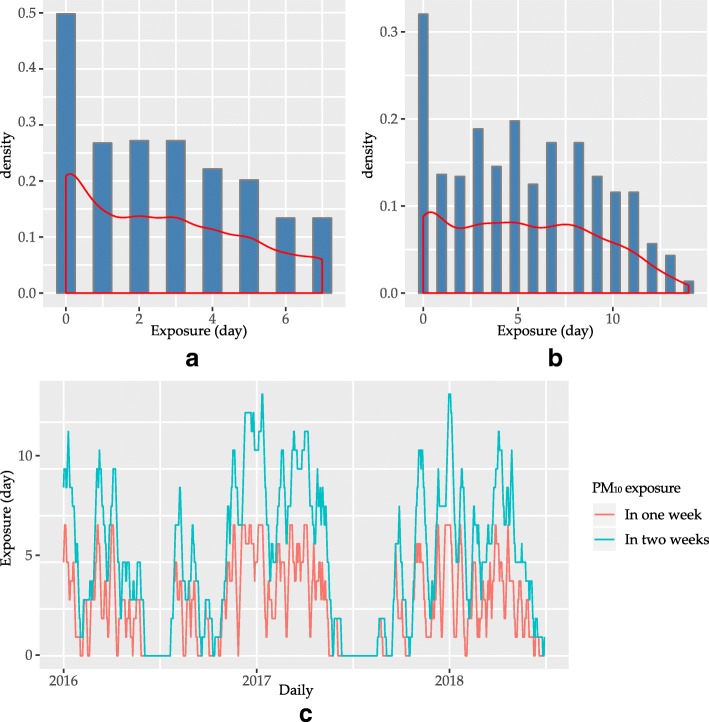
Distribution and exposure level to PM_10_.Panel (**a**): Distribution of exposure days of PM_10_ in one week before the exacerbation and the density curve; Panel(**b**): Distribution of exposure days of PM_10_ in two weeks before the exacerbation and the density curve; Panel(**c**): Exposure days of PM_10_ in one week (red line) and in two weeks (blue line) before the exacerbation

The summary statistics of environmental variables were summarized in Table 2. During the study period, the daily levels of PM_2.5_ ranged from 6 to 110 μg/m^3^ with an annual mean of 27.44 μg/m^3^. The mean PM_2.5_ concentration was 2.16% lower than the Grade II Annual PM_2.5_ Standard of CNAAQS (35 μg/m^3^), but 2.7 times higher than the annual average PM_2.5_(10 μg/m^3^) in WHO guideline. Daily levels of PM_10_ranged from 11 to 141 μg/m^3^ with an annual mean of 47.66 μg/m^3^. The exposure of PM_2.5_ for the cohort ranged from 0 to 7 days in one week before the exacerbation, and 0 to 14 days in two weeks before the exacerbation(Fig. 2). The exposure of PM_10_for the cohort ranged from 0 to 7 days in one week and 0 to 14 days in two weeks (Fig. 3). The average exposure to PM was 2 days in one week and 4 days in two weeks for PM_2.5_, and 3 days in one week and 6 days in two weeks for PM_10_.

**Table 2:** Overview of environmental variables in Xiamen

	Mean	SD	Minimum	First quartile	Median	Third quartile	Maximum
PM_2.5_(μg/m^3^)	27.44	14.535	6	17	24	35	110
PM_10_(μg/m^3^)	47.66	22.779	11	31	43	60	141
Temperature(°C)	21.30	6.22	3.9	15.97	22.05	27.12	31
Precipitation(mm)	4.07	12.76	0	0	0	0.9	172.7
Wind speed(m/s)	2.68	1	2	2	2.5	3.2	9.6

As for the weather conditions during the study period, the average daily temperature ranged from3.9–31 °C (annual average 21.3 °C) during the study period. The average precipitation ranged from 0 to 172.7 mm (annual average 4.07 mm). And the average wind speed ranged from 2 to 9.6 m/s (annual average 2.68 m/s).

### PM exposure versusRisk of exacerbation

**Fig. 4:**
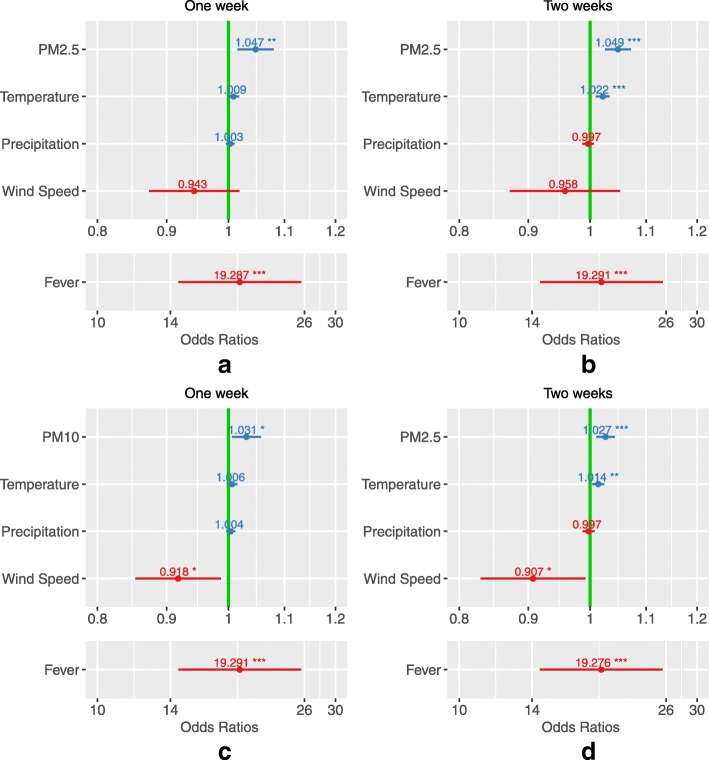
Odds ratiosof asthma exacerbation estimated for the exposure days to PM_2.5_ and PM_10_.Panel (**a**): Exposure days to PM_2.5_ within one week. Panel(**b**): Exposure days to PM_2.5_ within two weeks. Panel(**c**): Exposure to PM_10_ within one week. Panel(**d**): Exposure to PM_10_ within two weeks

The exposureto PM_2.5_ in one week (Standardized OR = 1.091; 95% CI: [1.029, 1.157]; *p* = 0.003) and two weeks (Standardized OR = 1.161; 95% CI: [1.084, 1.243];*p* < 0.001) were both significantly associated with higher risk of asthma exacerbation (Fig. 4, Table 3a). And the effect of PM_2.5_ exposure in two weeks was more severe than the exposure in one week.

**Table 3:** **a.** Odds ratios of asthma exacerbation for exposure days to PM_2.5_. **b.** Odds ratios of asthma exacerbation for exposure days toPM_10_

Variable	OR	Standardized OR	%increase	Standardized 95%CI	*P*-value
One-week-model					
PM_2.5_ Exposure (day)	1.047	1.091	9.10	[1.029, 1.157]	0.003**
Temperature (°C)	1.009	1.049	4.89	[0.994, 1.106]	0.079
Precipitation(mm)	1.003	1.020	1.96	[0.975, 1.066]	0.397
Wind Speed(m/s)	0.943	0.966	−3.44	[0.922, 1.011]	0.137
Fever	19.287	2.401	140.15	[2.208, 2.612]	< 0.001***
Two-week-model					
PM_2.5_ Exposure(day)	1.049	1.161	16.06	[1.084, 1.243]	< 0.001***
Temperature (°C)	1.022	1.125	12.52	[1.056, 1.199]	< 0.001***
Precipitation(mm)	0.997	0.984	−1.57	[0.941, 1.030]	0.492
Wind Speed(m/s)	0.958	0.979	−2.08	[0.935, 1.025]	0.370
Fever	19.291	2.402	140.16	[2.208, 2.612]	< 0.001***
One-week-model					
PM_10_ Exposure(day)	1.031	1.071	7.12	[1.013, 1.132]	0.015*
Temperature (°C)	1.006	1.035	3.53	[0.984, 1.090]	0.184
Precipitation(mm)	1.004	1.024	2.40	[0.977, 1.073]	0.323
Wind Speed(m/s)	0.918	0.950	−5.01	[0.909, 0.992]	0.021*
Fever	19.291	2.402	140.16	[2.208, 2.612]	< 0.001***
Two-week-model					
PM_10_ Exposure(day)	1.027	1.106	10.64	[1.042, 1.175]	< 0.001***
Temperature (°C)	1.014	1.079	7.93	[1.020, 1.142]	0.008**
Precipitation(mm)	0.997	0.989	−1.15	[0.943, 1.036]	0.633
Wind Speed(m/s)	0.907	0.954	−4.65	[0.913, 0.996]	0.033*
Fever	19.276	2.401	140.11	[2.208, 2.611]	< 0.001***

*P < 0.05, **P < 0.01, *** *P* < 0.001

Just like PM_2.5_, PM_10_ exposure during one week and two weeks showed a significant increase in the risk of asthma attacks. Each incremental day of exposure increased the risk of asthma onset by 7.12% (*p* = 0.015; 95% CI: [1.3, 13.2%], in one week) and 10.64%(*p* < 0.001; 95% CI: [4.2, 17.5%], in two weeks) (Table 3b).

As for weather conditions, temperature and wind speed had significant effect on asthma exacerbation. Rise of temperature increased the risk of asthma exacerbation, and increase in wind speed reduced the risk of asthma exacerbation. When exposed to PM_2.5_, the standardized OR of the temperature during one week was 1.049 (*p* = 0.079). The standardized OR of the temperature during two weeks was 1.125 (*p* < 0.001) (Table 3a). When exposed to PM_10_, the standardized OR of the temperature during two weeks was 1.079 (*p* = 0.008) (Table 3b). In addition, when exposed to PM_10_, the standardized OR of wind speed in one week was 0.950 (*p* = 0.021), and the standardized OR of wind speed in two weeks was 0.954 (*p* = 0.033) (Table 3b).

Fever had a significant effect on asthma exacerbation. When exposed to PM_2.5_ for two weeks, the standardized OR of fever was 2.402 (*p* < 0.001), and as for PM_10_, the standardized OR was 2.401 (*p* < 0.001).

### Association between PM exposure and disease control rate of childhood asthma

**Fig. 5:**
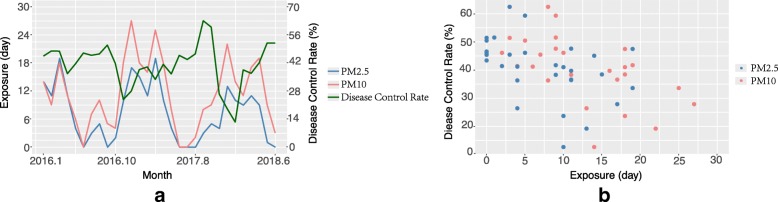
The association between PM exposure and DCR for childhood asthma. Panel (**a**):Time series of PM and DCR for childhood asthma during the study period. Panel (**b**): Distribution of PM exposure and DCR. PM_2.5_ (blue) and PM_10_ (red) were indicated

During the whole period, the exposure of PM_2.5_ and PM_10_ was higher in winter and lower in summer, while the control rate peaked in summer and was the lowest in winter (Fig. 5a). With the increase of days of PM exposure, the control rate showed a downward trend (Fig. 5b).

**Fig. 6:**
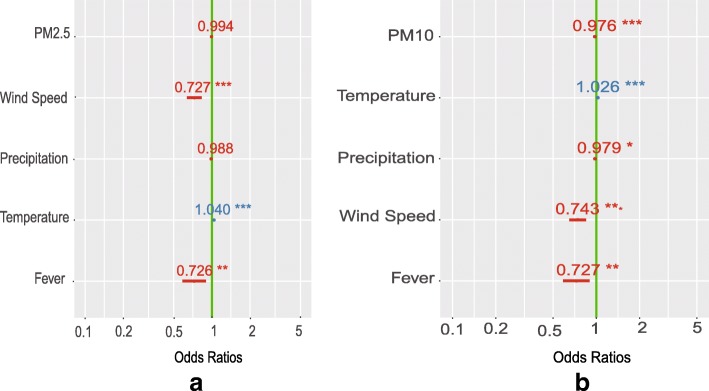
Odds ratios of each increasing day of exposure to PM_2.5_(**a**) and PM_10_(**b**) on DCR of childhood asthma

Among the 3443 returning patients, PM_2.5_exposure did not affect the control rate (*p* = 0.347, Fig. 6a, Table 4),however exposure to PM_10_ had a negative effect on childhood asthma control rate (Fig. 6b, Table 4),as each increasing day of exposure to PM_10_ reduced the odds of childhood asthma control by 15.18% (standardized OR 0.848;95%CI: [0.786,0.915], *p* < 0.001). Fever was associated with the decrease of DCR (standardized OR of PM_2.5_ was 0.923 and standardized OR of PM_10_ was 0.924).

**Table 4:** Odds ratios of asthma control for exposure days toPM_2.5_ and PM_10_

Variable	OR	Standardized OR	%increase	Standardized 95%CI	*P*-value
PM_2.5_					
Exposure(day)	0.994	0.959	− 4.10	[0.879, 1.046]	0.347
Temperature (°C)	1.04	1.256	25.56	[1.155, 1.365]	< 0.001***
Precipitation(mm)	0.988	0.962	−3.84	[0.910, 1.015]	0.159
Wind Speed(m/s)	0.727	0.883	−11.72	[0.837, 0.931]	< 0.001***
Fever	0.726	0.923	−7.67	[0.876, 0.974]	0.003**
PM_10_					
Exposure(day)	0.976	0.848	−15.18	[0.786, 0.915]	< 0.001***
Temperature (°C)	1.026	1.16	16.02	[1.079, 1.248]	< 0.001***
Precipitation(mm)	0.979	0.934	−6.62	[0.884, 0.987]	0.015
Wind Speed(m/s)	0.743	0.891	−10.95	[0.846, 0.938]	< 0.001***
Fever	0.727	0.924	−7.64	[0.876, 0.974]	0.003**

*P < 0.05, **P < 0.01, *** *P* < 0.001

## Discussion

Our study confirmed that the exposure to PM_2.5_and PM_10_ within one or two week sposed significant risk to exacerbation of childhood asthma in Xiamen, China. The risk of PM exposure was independent on the effects of other pollutants, weather conditions, or individual variation. In addition, our data suggested that the effect of exposure to PM lasted for at least weeks.

The association between PM exposure and the risk of asthma has been studied in different regions of the world and consensus have been reached that high exposure to PM causes increased risk of exacerbation and admission rate [34]. For example, one study conducted in Seattle, Washington suggested that for every 11 μg/m3 increase in PM_2.5_ concentration, the OR of childhoodasthma was 1.15 (95% CI: 1.08 to 1.23) [35]. An Australian survey which sampled 36,024 hospitalized patients with asthma showed that the impacts of PM_2.5_, NO_2_, PM_10_and pollen in the cold season on hospitalization for asthma were 30.2% (95% CI: 13.4 to 49.6%), 12.5% (95% CI: 6.6 to 18.7%), 8.3% (95% CI: 2.5 to 14.4%), and 4.2% (95% CI: 2.2~6.1%), respectively [36]. Taiwanese scholars used the open data of the government to investigate the air pollution in different urban models using time-stratified case crossover studies and conditional logistic regression analysis in 4237 hospitalized children with asthma in Taipei and Kaohsiung from 2001 to 2010 [37]. The results showed that the risk of hospitalization for childhood asthma was significantly correlated with air pollutants. After being adjusted by season, the air pollution in Kaohsiung City had greater impact on the hospitalization of childhood asthma than that in Taipei.

Although many studies addressed the effects of PM exposure on asthma risk in the long-term [17], less is known about the transient effects of PM exposure in the scale of weeks. Several recent studies address the effects of PM exposure on asthma exacerbation in short-term in Ningbo, Taipei, Seoul and Detroit [38–41]. According to these reports, the highest effect size of PM exposure on asthma exacerbation ranges from5 to 10 days. In order to accommodate the lagged effect we estimated the effect size for one week and two weeks of exposure, respectively. Our data confirm PM exposure as a risk factor to asthma exacerbation and the effect peaks at two weeks of exposure, which is consistent to prior studies. Moreover, our results suggest PM exposure has a negative effect on the disease control rate, which provided extra evidence for the hazardous impact of PM on childhood asthma. In an investigation of commuters, PM2.5 exposure was associated with lower FEV1% predicted among participants with below-median asthma control (3 h postcommute: -7.2 [95% CI = − 11.8, − 2.7]) [42]. A study in El Paso, Texas showed positive associations between Asthma Control Questionnaire (ACQ) scores and 96-h effects of PM_10_, PM_2.5_, black carbon, NO2 and ozone. In this study, the ACQ was used to evaluate asthma control [43]. Scottish scholars found that there is an exposure-response relationship between indoor PM_2.5_ concentration and poorer asthma control in children prescribed inhaled corticosteroids (ICS) [44]. The effect of PM_2.5_ in this study is reported after 5 days of exposure.

Prior studies use different measures to quantify the level of exposure to PM [39, 40]. In this study, we used the “Technical Regulation on Ambient Air Quality Index” (AQI, HJ 633–2012) [33] issued by Chinese government as an official standard classify air quality and use the total number of days of level 2 and 3 as a measure of exposure. The regional AQI is based on air-pollution measures from different sites and normalized for geological variations hence more accurate and comprehensive. In addition, the use of AQI makes our data directly applied to the regulation policies of pollution control and public health. There are other ways to measure the exposure to PM, such as the average concentration. Our results based on exposure days are consistent with and complementary to the prior studies.

PM exposure is not a stand-alone risk of asthma exacerbation. It has been previously shown that weather conditions, other environmental exposure, infections and self-management all contribute to the exacerbation of asthma. Our study is based on case-crossover design where each subject serves as its own control. Such a design can effectively remove inter-subject variations such as self-management. As for the weather conditions, temperature, barometric pressure and humidity are tightly correlated with each other, therefore, we kept only temperature to avoid collinearity. Co-morbid infections are not directly measured in the data we obtained but at the same time strongly affect the exacerbation of asthma. Therefore, we used surrogate variables such as the record of fever in the history of present illness.

To estimate the effect of PM exposure on DCR, we combine the uncontrolled and partly controlled subject into one group. The same classification is used in prior clinical studies of asthma exacerbation [31, 32]. Plus, around 20% of partly controlled asthma will develop into uncontrolled disease and has a risk of exacerbation (0.1%) [45, 46].

In spite of the growing concern over air pollution caused by PM, the hospitals and public health services in China still lack accurate regional assessment of the risk posed by PM exposure, which is required for risk management and preventative measures. The resultsofour study provided basis for preventative and clinical management of the exacerbation risk of asthma. In particular, we also described a method based on case-crossover design that can apply to other regions of the country.

Real-world evidence (RWE) has become increasingly important in medical and epidemiological research. Our study based on information extracted from local EHR database provides a plausible pipeline to address environmental risk factors using RWE, which enables more accurate estimate of the effects in large population. On the other hand, unknown bias factors can confound the analysis based on RWE, therefore we have considered all possible covariates. More importantly, the case-crossover design is based on self-control, thus, less affected by sampling biases.

Finally, the biological mechanism of the toxicity of PM is not fully elucidated inhuman. However, many studies confirm that the toxicity of PM is related to the immunogenicity and the consequential immune responses using cell line and animal model [47]. In OVA-sensitized mice, exposure to PM promote the proliferation of peribronchial lymph nodes and the activation of T-help cell subtype 2 which provokes inflammation in airway [48, 49]. Other studies suggest exposure to PM result in an increment of both neutrophils and eosinophils [50]; it also causes imbalance activities of Th1/Th2 through the activation of TNF- α and suppression of INF-γ [51, 52]. Moreover, prior studies also demonstrate that exposure to PM affect with the activities of monocytes and macrophages [53]. A number of pathological changes, such as inflammatory cell infiltration, bronchial smooth muscle thickening, and bronchial mucosal injury are observed following the exposure to PM [54]. More recent study shows that certain transcription factors, such as Toll-like receptor and nuclear factor-erythroid 2-ralated factor 2(Nrf2) signaling pathway are involved in the inflammatory responses in the airway of asthmatic mice [55].

The physicochemical property of PM varies substantially due to the source of pollutant as well as climate. It is still not clear what is the exact molecular basis underlying the toxicity of PM. Our results are constrained to the local conditions in Xiamen and may differ from other regions due to the different chemical features of PM. To address the question, systematic chemical description of the PM is needed in future study.

## Conclusions

This study assessed the short-term effects of air pollution and weather conditions on childhood asthma exacerbation and control rate in Xiamen. We confirmed that short-term exposure to PM for one or two weeks increased the risk of exacerbation in asthmatic children and compromises the disease control rate. Our study provides epidemiological data for formulating environmental health policy and clinical prevention of asthma in children. Our findings reaffirmed the necessity of preventive care for asthma susceptible population according to environmental conditions.

## Additional files

Additional file 1: The enrollment criteria of patients in the study. (DOCX 15 kb)

Additional file 2: Assessment of disease control of asthma for children below and above 6 years old. (PDF 343 kb)

## Abbreviations

ACQ: Asthma Control Questionnaire
AQI: Air quality index
CCO: Case-Crossover
DCR: Disease control rate
EHR: Electronic health record
ICS: Inhaled corticosteroids
Nrf2: Nuclear factor-erythroid 2-ralated factor 2
OR: Odds ratio
PM: Particulate Matter
RWE: Real-world evidence
