# Supplementary material for: Effects of particulate matter (PM) on childhood asthma exacerbation and control in Xiamen, China
Source: BMC Pediatr. 2019 Jun 13;19:194. doi: 10.1186/s12887-019-1530-7 (PMC6563520; doi:10.1186/s12887-019-1530-7)
Supplement: Supplementary file 1 — The enrollment criteria of patients in the study. (DOCX 15 kb) [file 12887_2019_1530_MOESM1_ESM.docx]

**Supplementary Table 1.** The enrollment criteria of patients in the study[1].

| Inclusive criteria | Symptoms and signs | 1. Respiratory symptoms such as wheezing, shortness of breath, chest tightness or cough, often triggered by contact with allergen exposure, changes in weather, physical and chemical irritants, infections, exercise and so on. | The diagnosis of asthma is based on all the symptoms and signs, and any of the confirmed variable airflow limitation. |
| --- | --- | --- | --- |
|  |  | 2. During the exacerbation, expiratory wheezing on auscultation occur and expiratory is prolonged. |  |
|  |  | 3. The above symptoms and signs can be relieved by treatment or automatically. |  |
|  | Confirmed variable airflow limitation | 1. Positive bronchodilator reversibility test (after inhalation of bronchodilator, FEV1 increased by >12% and >200 ml from baseline) |  |
|  |  | 2. Positive bronchial challenge test |  |
|  |  | 3. Average daily diurnal peak expiratory flow (PEF) variability >10%, or weekly PEF variability >20%. |  |
| Exclusive criteria | | Wheezing, shortness of breath, chest tightness or cough caused by other diseases. |  |

**Supplementary Table 2.** Assessment of disease control of asthma for children below and above 6 years old[1].

| Children aged 6 years or older |  |
| --- | --- |
| 1. daytime symptoms more than twice/week | None of the items is classified as well controlled asthma; 1-2 items as partly controlled asthma; 3-4 items uncontrolled asthma |
| 2. night waking due to asthma |  |
| 3. reliever needed for emergency more than twice/week |  |
| 4.activity limitation due to asthma |  |
| Children under 6 years old |  |
| 1. daytime symptoms lasting at least several minutes more than once/week | None of the items is classified as well controlled asthma; 1-2 items as partly controlled asthma; 3-4 items uncontrolled asthma |
| 2. night waking or cough due to asthma |  |
| 3. reliever needed for emergency more than once/week |  |
| 4. activity limitation (less running/playing compared to other children, easy to fatigue when walking/playing) due to asthma |  |

1. The Subspecialty Group of Respirology. The Society of Pediatrics, Chinese Medical Association Guidelines for the diagnosis and prevention of asthma in children (2016). Chin. J. Pediatr. 2016. doi:10.3760/ema.j.issn.0578—1310.2016.03.003
