# Supplementary figures and images for: Effects of particulate matter (PM) on childhood asthma exacerbation and control in Xiamen, China
Source: BMC Pediatr. 2019 Jun 13;19:194. doi: 10.1186/s12887-019-1530-7 (PMC6563520; doi:10.1186/s12887-019-1530-7)

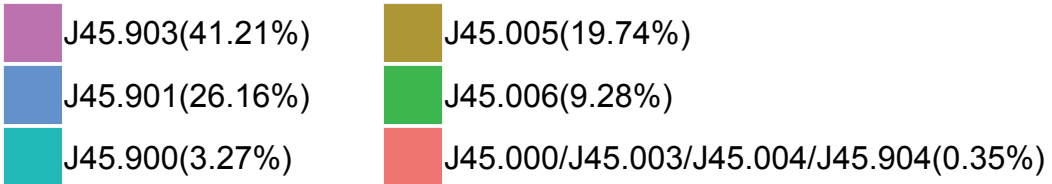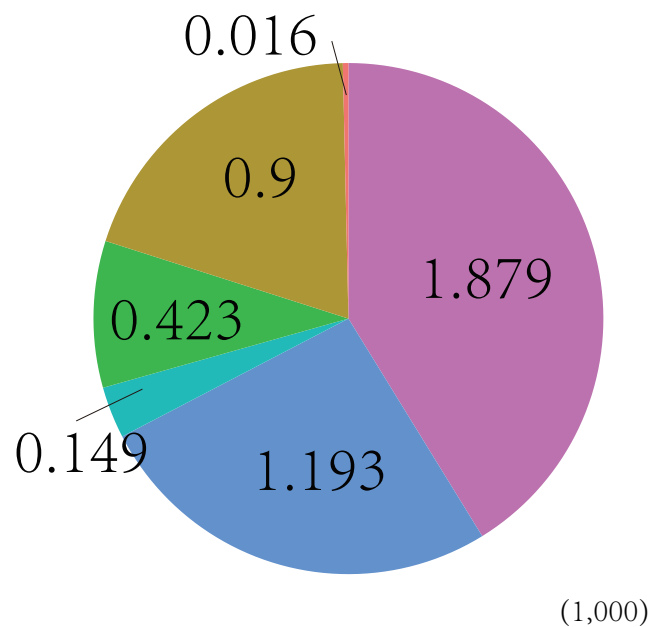

(a)

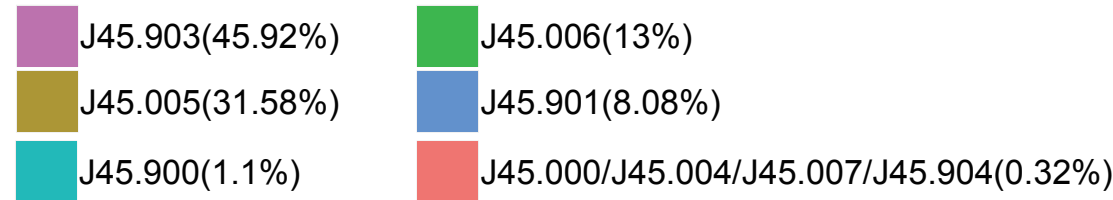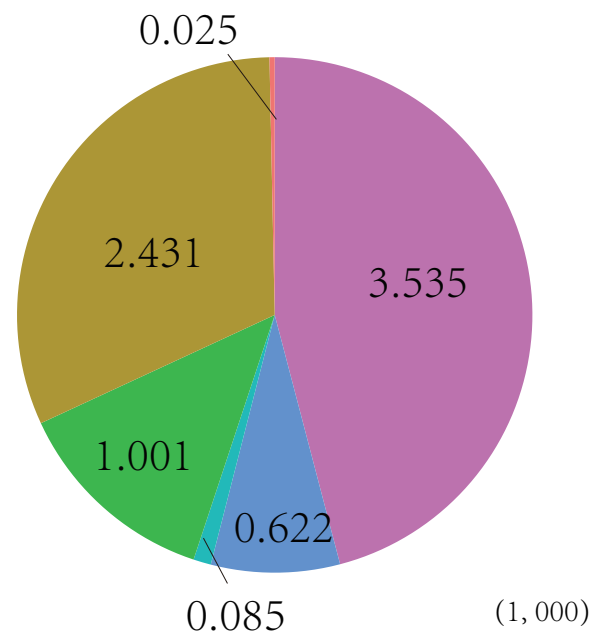

(b)

Supplement: Supplementary file 2 — Assessment of disease control of asthma for children below and above 6 years old. (PDF 343 kb) [file 12887_2019_1530_MOESM2_ESM.pdf]
